# Residents’ experiences of encounters with staff and communication in nursing homes during the Covid-19 pandemic: a qualitative interview study

**DOI:** 10.1186/s12877-022-03627-x

**Published:** 2022-12-12

**Authors:** Elisabet Eriksson, Katarina Hjelm

**Affiliations:** 1 Department of Public Health and Caring Sciences, Uppsala University, Box 564, 751 22 Uppsala, Sweden; 2 Faculty of Health and Occupational Studies, University of Gävle, 801 76 Gävle, Sweden

**Keywords:** Communication, Old people, Nursing homes, Covid-19 pandemic, Nursing

## Abstract

**Background:**

The Covid-19 pandemic and associated visiting restrictions have challenged communication with others for older people residing in nursing homes.

**Aim:**

The aim of this study was to explore residents’ experiences of encounters and communication with staff and relatives and friends during the Covid-19 pandemic.

**Design:**

An exploratory study with an inductive approach.

**Methods:**

Semi-structured telephone interviews with 16 Swedish nursing home residents were conducted. Data were analyzed using qualitative content analysis; the study reports according to the COREQ checklist.

**Results:**

Three main categories emerged: (1) Mixed feelings and experiences of encounters with nursing staff, (2) Adapting to hampered communication and finding strategies to overcome language barriers, and (3) Facing pandemic restrictions and living an adjusted life. Nine subcategories are reported within these categories. Residents mainly reported good encounters with staff and receiving the care they needed, but unhelpful encounters were also reported. To manage communication with staff with limited Swedish language skills, residents developed several strategies. During the visiting ban, residents felt secure but also lonely. Staying connected with the outside world required residents to use the phone and handle other digital aids, such as video calls, but lack of technical skills among staff hindered frequent use of video calls.

**Conclusion:**

This study highlights how residents can feel safe in extreme situations, but increased competence, including digital literacy and language skills, among staff is required. Care providers should provide relevant information to residents and staff and employ competent staff. Findings indicate that communication plans need to include enabling communication for residents both within and outside nursing homes, taking staff language skills into consideration.

**Supplementary Information:**

The online version contains supplementary material available at 10.1186/s12877-022-03627-x.

## Background

Older people (hereafter called residents) in nursing homes (NHs) develop relationships with staff unintentionally by simply trying to have a life at the NH, although few residents report close relationships with staff [1, 2]. Residents may have difficulties to communicate, as some older people have impaired hearing, vision, or cognitive ability. Due to increased global migration [3] and lack of care staff in many countries, NH residents, their relatives and care staff may have different native languages and cultural backgrounds [4–6]. Of Sweden’s 10.4 million inhabitants, 20% were born abroad and about one third of assistant nurses working in home care and in NHs are foreign born [7, 8]. Although residents are aware of the cultural diversity among NH staff and may find it appealing, residents also perceive language dissimilarities as a challenge when communicating with staff [5, 6].

During the Covid-19 pandemic, residents experienced loss of in-person contact with family members, friends, and other social ties for a long period of time due to restrictions such as social distancing and restraining order. To keep in contact with others, residents had to rely on Information and Communication Technologies (ICTs). Studies have shown that residents’ use of phone calls, video calls and chats increased during the pandemic [9–13], but the accessibility of ICTs varied across NHs. Moreover, some residents had to rely on staff being able to assist them in using ICTs, and staff shortages were common during the pandemic [9–11].

The situation for NH residents received special attention during the Covid-19 pandemic [14]. Due to their old age, NH residents belong to one of the groups that are at increased risk of severe symptoms of a Covid-19 infection [15]. Several factors make the older people susceptible to infection, such as congregate living, difficulty following the recommendations regarding hand hygiene and physical distancing, exposure to staff with unknown Covid-19 status, and comorbidities. In the early phase of the pandemic, the Swedish National Board of Health and Welfare (NBH) reported place of death for people aged 70 and older who had died due to Covid-19, showing that 979 of 1877 deaths due to Covid-19 occurred in NHs [16]. Moreover, even later in the pandemic, most deaths due to Covid-19 (18,484 of 20,753) occurred among people aged 70 years and older [16]. With the goal of preventing Covid-19 infections, Sweden, like many other countries, followed the recommendation that the number of visitors should be reduced and highly restricted in healthcare settings [17]. Therefore, the Public Health Agency of Sweden introduced national restrictions on visiting older people in NHs from April 1 to September 30, 2020 [18]. Thereafter, NH management were encouraged to set up visiting practices to minimize infection transmission. These practices included wearing personal protective equipment (PPE), keeping physical distance from each other, and using plexiglass between visitors and residents. Thus, residents’ social contacts and communication were limited to care staff and other NH residents, and the relationship between residents and care staff became more significant. Prior research has reported loneliness among NH residents as a consequence of visiting restrictions [19, 20]; although the reported perceived loneliness among NH residents differs [21, 22]. Relatives of NH residents have further reported negative change in mood and residents becoming more withdrawn during the pandemic [23, 24]. An interview study among relatives of NH residents in the same geographical region as the present study also reported residents being lonelier during the visiting ban [25]. Additionally, residents have died alone without relatives or staff present [26]. During the pandemic, NH residents have lived under extreme circumstances and experienced social isolation for shorter or longer periods of time. In addition, their physical contacts have often been limited to healthcare staff during a period of high staff turnover. No previous study exploring residents’ experiences of encounters and communication, including communication with staff with limited skills in residents’ native language, was revealed in the literature review. The aim of the present study was to explore residents’ experiences of encounters and communication with staff and relatives and friends during the Covid-19 pandemic.

## Methods

### Design

A qualitative explorative study design was chosen to acquire in-depth knowledge about a specific topic in an area that has previously scarcely been studied [27]. Semi-structured interviews were selected to collect data, as they allow participants to speak freely within specific areas and enable follow-up questions that probe deeper into respondents’ thoughts.

### Study setting

The study was conducted in a county including eight municipalities and approximately 380,000 inhabitants in central Sweden. In the county, NHs are run by the municipalities and by private companies. In Sweden, NHs usually have common dining rooms and living rooms, but each resident has their own separate room. The resident’s room may have simpler kitchen equipment for preparing meals. Couples may have larger rooms suitable for two people. For the public, tracing of the transmission of Covid-19 was not required at the time of writing. However, tracing of Covid-19 transmission is still required in inpatient care and care activities targeting people with a high risk of becoming seriously ill from Covid-19, such as NH residents [28].

### Sample and procedure

A purposive sampling strategy was used. The characteristics of the 16 residents who participated are presented in Table 1. All were born in Sweden, had various educational backgrounds and occupations, and had experience of working as teachers in pre-school, primary school, language or piano, as a vice-chancellor, nurse, bus driver, self-employed, librarian, farmer, accountant, engineer, kitchen assistant, and seaman. Most residents had participated in choosing the NH they lived in (Table 1) and felt satisfied with their situation at the NH. They enjoyed participating in activities such as playing bingo, different entertainment, physiotherapy, and outdoors activities. However, residents also reported that they had no one to talk to at the NH because other residents were older, had impaired hearing, or suffered from dementia and did not recognize them. One man said that only talking to female residents was not fun, as he was the only man on his ward. The languages they understood apart from Swedish were English, German, and French.

**Table 1:** Characteristics of the study population, residents

Variable	Residents ***N*** = 16
Age (years)^a^	82.5 (71–97)
**Gender** (n)	
Male	5
Female	11
**Country of birth** (n)	
Sweden	16
**Level of education** (n)	
Primary school (< 9 years)	8
Upper secondary school (≥ 9–12 years)	3
Education at university level < 2 years	1
Education at university level ≥ 2 years	4
**Family status** (n)	
Married	2
Unmarried	2
Divorced	3
Widow/widower	9
**Number of languages reported to be understood** (n)	
≤ 2	11
3–4	5
**Languages older people reported they could understand** (n)	
Swedish	16
English	7
German	4
French	4
Latin	1
**Had participated in choosing care home for older people** (n)	
Yes	13
No	2
By chance	1
**Wish to stay at another care home for older people** (n)	
Yes	2
No	13
Uncertain	1
**Number of years at the care home for older people**	
≤ 2	12
3–4	3
≥ 5	1

^a^Values are Median (Range)

Some residents described their health status as good, while others reported having a chronic disease, e.g., diabetes and kidney disease, and others reported problems like hearing difficulties, impaired vision, and walking difficulties. Three residents reported having had a Covid-19 infection (data not shown).

Approval to conduct the study was obtained from operations managers for NHs institutions in eight municipalities. Thereafter, the principal investigator (PI; a nurse skilled in medical care (first author)) contacted the first-line managers at the NHs (*n* = 67) by email and/or phone and informed them about the study. Six first-line managers declined to participate due to the strained situation at the NH. Nine NHs in three municipalities participated; seven were public NHs and two were privately owned. Then, the first-line managers informed care and administrative staff about the study, and in some NHs, the PI participated in digital staff meetings to provide information. Caring staff and first-line managers informed residents about the study and provided the PI with residents’ names and phone numbers. Caring staff and first-line managers asked residents living in nursing wards and not from wards especially attributed to dementia care who were able to speak Swedish and participate in telephone interviews. The PI contacted 21 residents to schedule a time for the interview. Five residents could not participate due to impaired hearing (*n* = 2), deteriorating health (*n* = 2), and wrong contact information (*n* = 1).

### Data collection

Semi-structured telephone interviews, held in Swedish, were conducted between November 2020 and December 2021 by two female registered nurses and researchers experienced in medical care, as well as with previous experience of conducting interviews. All interviews except three were conducted by EE. Interviews by telephone were the only possible data collection method due to visiting restriction bans, although this is not the preferred method for communicating with older people [29]. However, the interviews worked well, and all questions were covered. The interviews were recorded on a MP3-player, lasted between 20 and 53 minutes, and were transcribed verbatim. The interview guide was developed by the research team (EE, KH experienced researcher and professor with expertise in qualitative research in nursing), based on relevant literature, and covered the following areas: (1) participants’ sociodemographic background data, (2) communication with staff at the NH, including communication with staff with limited Swedish language skills, (3) perceived encounters with staff (4), how the Covid-19 pandemic may have affected their communication/relationship with their family members and friends, and (5) their experiences of restrictions during the Covid-19 pandemic. To encourage participants to further explain their experiences, additional follow-up questions were asked such as ‘Could you give an example of how you communicated with your family during the visiting restrictions?’ The data collectors had no relationship with participants or the management at the NHs prior to data collection.

### Data analysis

Collection and analysis of data proceeded simultaneously until no new information was added in the analysis by including new informants (Patton 2015). The interviews were transcribed verbatim (by a professional secretary) and thereafter read through by the PI to get an overview of the content; each interview was considered a unit of analysis. Qualitative content analysis with an inductive approach was used (Patton 2015). Text and phrases related to the study aim were identified, and meaning units were marked in the transcripts. The meaning units were thereafter coded, and codes were compared for similarities and differences between them. Groups of codes sharing mutual attributes were organized into subcategories and categories and labelled. The first author performed the data analysis and both authors coded and compared two transcripts. Finally, the second author provided regular input through discussions during the analysis. Open Code, a computer application that assists with labelling and organizing data, was used [30].

### Rigor

Credibility was strengthened as both authors reflected on and discussed the content and code labels, subcategory, and category names [27]. Any disagreements regarding the coding and labelling of subcategories and categories were solved through discussions. To Further strengthen the credibility of the study, a purposive sampling procedure was used, the goal being to include residents who varied in age and gender and who lived at different NHs [27]. To enhance dependability, an interview guide was used to ensure that participants were asked the same questions (Additional file 1). Descriptions of the participants’ characteristics, information on the setting, and the data analysis are provided to increase the transferability of the results. By exemplifying each presented category with quotes from the participants’ responses, confirmability was achieved. The Consolidated Criteria for Reporting Qualitative Research (COREQ) were followed [31].

### Ethical considerations

The present study was approved by the Swedish Ethical Review Authority (Reg. no. 2020–03636 and 2020–07193) and implemented in accordance with the Helsinki Declaration [32], including written informed consent. Transcripts were deidentified and digitally stored in a space intended for research data, accessible only to the researcher.

## Results

The data analysis resulted in three main categories: mixed feelings and experiences of encounters with nursing staff, adapting to hampered communication and finding strategies to overcome language barriers, and facing pandemic restrictions and living an adjusted life. These and the nine subcategories are presented in Table 2, and are further described in the following section, exemplified using illuminative quotations when applicable.

**Table 2:** Overview of categories and subcategories

Category	Subcategories
Mixed feelings and experiences of encounters with nursing staff	o Experiencing both kind and unkind encounters with nursing staff o Which staff will one encounter in a situation with high staff turnover o Experiencing inconvenience and discomfort due to lack of competence among staff o Varied experiences of delivered care
Adapting to hampered communication and finding strategies to overcome language barriers	o Understanding for language difficulties o Facing impaired communication o Striving to be understood
Facing pandemic restrictions and living an adjusted life	o Feeling secure but lonely and locked in o Keeping in contact with persons outside the NH during visiting restrictions

### Mixed feelings and experiences of encounters with nursing staff

Residents reported both gentle and unfriendly encounters with staff and that high staff turnover led to concerns about who would be caring for them. Overall, residents were satisfied with the care they received, but shortcomings in the care they received were also reported.

#### Experiencing both kind and unkind encounters with nursing staff

Overall, residents reported staff being kind to them. Staff were described as accommodating, glad, helpful, friendly and very nice. Being able to laugh and joke with staff was appreciated. Some residents described their relationship with the staff as relaxed, and one participant with no living relatives described a very close relationship with the staff: ‘and we can laugh together, it’s like I belong with the staff members somehow’ (Resident 16). However, residents also described encounters with staff using more neutral terms, saying they had nothing to complain about, that they had more contact with some staff members than others and that the relationship depended on the different personalities. More negative experiences were also reported by some residents, for example that not all staff were perceived as angels and that they were being treated as more disabled or as having dementia. Some staff were described as difficult to cooperate with, and residents sometimes firmly corrected staff: ‘I’m with it enough mentally that I can refuse things. And then it works out eventually. Because I just tell them straight out that it shouldn’t be like that’ (Resident 1). However, residents also reported that they did not want to complain to the manager about staff behavior. Residents’ encounters with foreign-born staff were also described as mixed, with some residents having good experiences while others were more skeptical.

#### Which staff will one encounter in a situation with high staff turnover

High turnover among staff resulted in residents feeling uncertain and worried about which staff they would encounter. Residents described that new and temporary staff created problems for them, for example that they had to explain themselves more in detail; it was difficult to remember their names, and it could get confused during mealtimes: ‘then we get new staff members who don’t know us at all. It can get pretty confusing, especially in the dining room’ (Resident 11). High staff turnover was described as a disappointment for everyone, for the residents but also for staff who were trying to make the best of the situation. Residents expressed their desire for more staff continuity.

#### Experiencing inconvenience and discomfort due to lack of competence among staff

Some residents reported low competence among staff, for example knowledge about how to put dressing on a simple wound. Others reported having been served food they were allergic to and having become ill when they ate the wrong food. Residents further mentioned that some staff did not know what to do and that they could ignore the care needs of other residents. They reported that staff needed basic knowledge and information in some areas, e.g., basic nursing, wound care, and a longer introduction period when they started working at the NH: ‘Because I don’t think they get enough information when they come here and start working’ (Resident 12). Regarding technical support for assistance with TV, tablets and computers, staff were often unable to help residents: ‘At this age, we’re not so accustomed to TVs and telephones and all those things. I wish there was someone here at the nursing home who was available and could help us’ (Resident 6).

#### Varied experiences of delivered care

The residents reported mixed experiences of care at the NHs. Many were satisfied with the care they received: ‘I get everything I need, and a bit more’ (Resident 1). Others reported receiving the care they needed: ‘You get help with what you need help with’ (resident 10), while some said they did not receive the care they wanted. Staff shortages and lack of time for staff to assist residents were also reported. Several residents wished that staff had enough time to sit down and talk with them: ‘there are a few hours between mealtimes and … when there is nothing special to be done. Never that they [staff] come in and talk a little’ (Resident 11), another resident said: ‘they talk, while they do things. But they don’t talk just like that, just sitting and talking’ (Resident 10). Other residents wished staff to take them out for walks or arrange social activities at the NH. One resident who needed help to empty a urinary catheter described waiting a long time for staff to assist her and the pain she experienced: ‘I waited for almost an hour. It’s painful, you know, because the bag is full and the pressure builds up, so several times I’ve been on the verge of crying, it’s true‘(Resident 7).

### Adapting to hampered communication and finding strategies to overcome language barriers

The residents reported that communicating with staff with limited Swedish language skills was very common, and they accepted that misunderstandings could occur. However, impaired communication was described as irritating, as things sometimes did not turn out the way they wanted. The residents used several strategies to make themselves understood.

#### Understanding for language difficulties

Foreign-born staff were very common at the NHs, and residents displayed their understanding that both permanent and temporary staff had limited Swedish language skills. Residents mentioned that, due to lack of staff, managers had to employ people with limited Swedish language skills. Some residents compared these communication barriers with being abroad: ‘naturally, I’ve been abroad … so I understand that if you don’t know the language, things sometimes go wrong’ (Resident 3). Furthermore, among residents an accepting attitude toward staff with limited language skills was evident: ‘I have to consider that it’s the same when I speak with them, that they don’t always understand me right away either’ (Resident 16). However, residents also reported that foreign-born staff had sufficient language skills and that there were no misunderstandings due to language.

#### Facing impaired communication

According to the residents, limited language skills among foreign-born staff was common and that staff needed to learn Swedish: ‘Their Swedish simply isn’t good enough. They don’t understand what you mean’ (Resident 2). Furthermore, they reported that communication was problematic and took time, especially when several foreign-born staff were working at the same time: ‘When there are several staff with a foreign language working at the same time, then it can be a bit of a problem’ (Resident 3). Communication was hampered when staff did not know the name of food, could not express their thoughts in words or participate in discussions:’ You can never discuss anything with them...They have not enough experience with this....If you start having some kind of philosophical reasoning, then their vocabulary runs out’ (Resident 1).

Residents reported that staff pronounced words incorrectly, could not read compound words and that more nuanced communication did not work. This was irritating for residents. Some reported that, due to lack of communication, things did not turn out the way they wanted, for example when taking a shower, putting on clothes, or during mealtimes. Furthermore, participating in social activities when foreign-born staff read books aloud was meaningless: ‘we have reading time in the afternoon, and then one of the new staff has to read, and they don’t even know what they’re reading …. They can’t read a story in an enjoyable way. That’s how it is.’ (Resident 12). Residents also described misunderstandings as minor, and that problems were resolved over time: ‘but they [the misunderstandings] aren’t anything you can’t survive’ (Resident 12). However, in some occasions communication was simply not possible ‘INTERVIEWER: So how does this [language skills] affect communication? ‘With some it goes well, and with some it doesn’t at all’ (Resident 2).

#### Striving to be understood

When talking to staff with limited knowledge of Swedish, residents reported that they spoke more slowly, listened more actively, and tried to understand them: ‘I try to replace words. I have lots of synonyms’ (Resident 5). They further mentioned using gestures or pointing at things they wanted or replacing words and phrases. To get things the way they wanted, residents could ask repeatedly and nag a bit when staff did not understand: ‘and if they don’t understand I just talk about it even more’ (Resident 8). Another strategy was to ask for help from permanent staff, but also to inform them that new staff needed assistance. Residents also reported sometimes having fun when they tried to understand each other and to teach foreign staff Swedish, if they were in a good mood. Nonetheless, residents wished staff members spoke Swedish better, saying it was not residents’ job to teach them Swedish. All residents tried to speak Swedish with staff as they did not have any other common language. The use of translating tools was not mentioned in the interviews.

### Facing pandemic restrictions and living an adjusted life

Pandemic restrictions came suddenly, and residents adjusted accordingly. Although they felt safe at the NHs during the pandemic, they described lonely days with few activities. During this period, residents mainly used phone calls to stay connected with their relatives and friends.

#### Feeling secure but lonely and locked in

Residents described how they received information about Covid-19 from the NH staff. The information provide was both good and poor, and residents also got information from TV, radio, and relatives. Some reported that NH first-line managers took the pandemic very seriously and provided them with information on how to protect themselves. Introducing restrictions, keeping distance, and using PPE helped residents feel safe at the NHs: ‘I’ve felt safe because they tell us what’s happening and how we should behave, so that we get through this’ (Resident 16).

The time with restrictions was described as boring. There were only a few activities they could participate in, such as reading, watching movies, playing bingo, taking part in physiotherapy, and listening to entertainment arranged outdoors. Several residents reported that they had spent days or weeks in their room, unable to see others during mealtimes: ‘It was no fun to suddenly not be able to eat meals with others … and say hello to them and everything’ (Resident 3). Others reported not being able hear what others said during mealtimes because they had to keep a distance. During the pandemic, residents tried to entertain themselves by reading books, listening to the radio and watching TV. However, the days became very long: ‘I have a TV and solve crossword puzzles … but some days are very long’ (Resident 9). Residents talked about feeling sad, lonely, and isolated during the pandemic, and some said they had lost faith in everything. Their hope for the future was to see their grandchildren, children, friends and to resume social activities. Only a few residents reported not having felt lonely, saying that the time with pandemic restrictions went better than expected. Communicating with staff who were wearing PPE was difficult for residents with impaired hearing.

#### Keeping in contact with persons outside the NH during visiting restrictions

To stay connected with relatives and friends, residents used phone calls, wrote and received letters, sent emails and a few had experiences of video calls: ‘Well, just the telephone. I usually say it was my lifeline’ (Resident 10). Further, residents described various ways of having physical contact, such as talking through the window or outdoors at a distance. To see their children and know they were healthy meant a great deal to the residents. However, visiting was also described as complicated, as their relatives had to book a time for the visit in advance. Grandchildren were not allowed to visit at NHs, and residents talked about having lost contact with them. Because children grow quickly, the residents missed seeing their development and feared that their grandchildren would be afraid of them once they could see each other in person: ‘she’s a very small child, she doesn’t know who I am. And so I just can’t start hugging her. That would scare her’ (Resident 3).

## Discussion

The results of present study provide unique insights into residents’ experiences of living in NHs during the Covid-19 pandemic by focusing on their encounters and communication with staff and people outside the NH. Overall, residents reported having good encounters with staff, but unhelpful encounters were also mentioned. High turnover among staff led to worries about which staff residents would encounter, as they were dependent on staff assistance. Residents received the care they needed, but some reported incidents of pain or lack of care related to staff not having the time or required competence to assist them. Moreover, residents had to manage communication with staff who had limited Swedish language skills, and they had developed several strategies for making themselves understood. In the already strained situation at NHs pre-pandemic – one characterized by low staffing of nurses and almost 25% of care staff being hourly employees – the pandemic restrictions implied new communication challenges regarding, for example, social distancing and staff use of PPE [33]. Residents with a hearing impairment reported difficulties when communicating with staff who were wearing masks. Residents felt safe during the ban on visiting NHs, but also lonely and bored, as almost all social activities had been cancelled. Staying connected with family and friends required residents to use the phone or ICT. Residents who were unable to handle digital aids, such as video calls, reported sometimes receiving assistance from staff, but lack of technical skills among staff hindered frequent use of ICT devices.

The present results revealed high acceptability and adaptability among residents with regard to living through extraordinary circumstances. Residents adapted to temporary staff during a time of high staff turnover. Nevertheless, it is noteworthy that some residents reported negative encounters with staff. Furthermore, residents reported sometimes feeling obliged to speak up on behalf of themselves or other residents and, in some cases, to report incidents to the NH manager. However, residents also mentioned that they did not want to complain to the manager about staff behavior. Low education and high age among the respondents may explain why some residents feared, or were not used to, reporting staff members’ unhelpful behavior to managers [1]. Lack of competence among care staff during the pandemic may explain staff members’ negative behavior toward residents [34]. Given that residents have a dependent relationship with staff, managers of NHs should ensure that staff with sufficient competence are available even in times of crisis. It has previously been emphasized that medical competence among staff needs to increase if the care system is to ensure residents’ safety [33, 35].

Residents reported understanding the situation of staff with limited language skills, although impaired communication created frustration and stress in everyday life. Although the respondents did know several languages (e.g., English, German, French), these did not match the languages spoken by foreign-born staff. These results indicate that care staff working at NHs in Sweden constitute a heterogeneous group. Thus, residents adapted and tried to speak Swedish with care staff who had limited skills in Swedish. Also, in accordance with previous research demonstrating that foreign-born nursing staff have difficulties participating in complex cross-cultural communications [36] and experience communication barriers [37], residents in the present study missed out on opportunities to have deeper discussions with foreign-born staff. Surprisingly, residents did not mention using any translating tools, like existing translation apps, or an interpreter. In (a country like) Sweden, one would expect technical solutions to be used more frequently than was found in the present study, indicating the need for future studies exploring the availability of language aids adapted to NHs settings and the barriers to using such aids. Instead, residents developed their own strategies for facilitating communication, for example using gestures. Use of non-verbal communication among NH residents has been reported previously [6]. However, when several staff with limited Swedish language skills were working the same shift, these strategies were not enough. Thus, residents were very vulnerable in these situations. However, according to residents’ reports, no physical harm resulted directly from communication barriers (but did potentially result from competence levels). Furthermore, it is possible that residents with severe dementia had a different experience or problems. Mangers employing staff with limited language skills in the native language need to ensure that staff improve their language skills, for example, through nurse-led programs, the goal being to enable fruitful communication between staff and residents [38].

In line with previous research [19, 20], the present findings demonstrate that residents felt lonely and locked in during the visiting ban. Nevertheless, residents felt safe at the NH when first-line managers informed them about Covid-19 and acted by initiating restrictions, keeping distance, and requiring staff to wear PPE. These results are interesting; they indicate that, in critical situations, when appropriate measures are taken, managers can help residents feel secure. This is an important lesson of the Covid-19 pandemic for nursing management. To reduce their loneliness and maintain contact with relatives, residents used phone calls, but also adapted to new communication channels, such as video calls. However, residents expressed their desire for more technical assistance from staff to enable both more frequent use and more advanced technical communication channels. The Covid-19 pandemic has highlighted the need for structures and organization that enable residents to communicate with relatives and others outside the NH. Clear communication plans need to be developed to prepare for future critical situations, such as when residents experience lock-downs at their NH. These plans need to ensure that residents have access to several ICTs, as video calls may bring residents greater satisfaction [39]. Furthermore, NHs need to teach staff the technical skills required to assist residents as well as ensure that staff can communicate in the language residents speak. Examples from this pandemic include structured tools for communication and care planning [40], training packages to support NHs in implementing risk management strategies [35], and courses in technological literacy for nurses [41], which could be offered to care staff working closest to residents. These actions can prevent residents from being both digitally and socially excluded [42] during a future crisis.

## STRENGHS and limitations

The present study provided valuable insights into older people’s experiences at NHs during the Covid-19 pandemic. However, the study has some limitations. Use of a qualitative study design does not enable generalization of the study results, but it does provide a deeper understanding of the participants’ experiences. Because phone interviews were conducted, some words were difficult to hear, but, overall, the phone interviews went well. However, residents with impaired hearing or cognitive function could not participate in the study. Thus, selection bias is possible [27]. Another limitation is that it is possible that residents felt pressured to participate, as they were recruited by NH staff. To avoid this, the interviewer informed the residents that participation was voluntary before asking the interview questions.

One strength is that the interviewers were trained and experienced in the interview process. Furthermore, the interviews were conducted in a manner that enabled rich knowledge to be generated. Although the target population was not involved in designing the study, one strength is that phone interviews were accepted by the residents.

## Conclusion

The present study contributes new knowledge on how experiences of safety in extreme situations can be upheld by providing information, introducing PPE and enabling fruitful communication both in and outside the NH. However, the study also reveals unmet needs among residents. Based on the study results, we conclude that translating tools were not used in cross-culture communications between staff and residents. Thus, our study also shed new light on the need to develop translation tools for NH settings – tools that are readily available and easy to use. To ensure that residents feel secure in extreme situations such as pandemics care providers and NH managers need to provide relevant information to residents, have capacity to take appropriate safety precautions and hire competent staff. Furthermore, communication plans need to include facilitating communication for residents both within and outside the NH, taking language skills among staff into consideration.

## Supplementary Information

**Additional file 1.**

## Data Availability

The datasets are not available from the corresponding author on request due to reasons concerning the protection of participants’ privacy and confidentiality.
