# Supplementary material for: Residents’ experiences of encounters with staff and communication in nursing homes during the Covid-19 pandemic: a qualitative interview study
Source: BMC Geriatr. 2022 Dec 12;22:957. doi: 10.1186/s12877-022-03627-x (PMC9744594; doi:10.1186/s12877-022-03627-x)
Supplement: Supplementary file 1 — Additional file 1. [file 12877_2022_3627_MOESM1_ESM.docx]

**Topic guide**

*Background data:*

How old are you?

Would you describe yourself as a woman, man or otherwise?

What is your country of birth?

How many years have you attended school?

What have you worked with?

Are you married, cohabiting, divorced, widow, widower or living alone?

What languages do you understand?

Have you participated in choosing your nursing home?

Do you wish to move to another nursing home?

How many years have you lived at this nursing home?

How are you doing at the nursing home?

What social activities are there that you can participate in?

Is there anything that is especially good / bad at the nursing home?

*Communication with staff at the NH, including communication with staff with limited language skills in Swedish:*

How does it work to speak with the staff?

Is it easy to speak / communicate with the staff? If not, can you tell me why?

Do staff members speak a language other than Swedish with you? Do you know which ones?

How do you communicate with staff if it is difficult to understand what they are saying?

Can misunderstandings arise if it is difficult to understand the staff, can you give an example?

How do you handle the situation when there is a misunderstanding?

How do you experience being cared for by staff born in another country?

*Perceived encounters with staff:*

How would you describe the relationship between you and the healthcare staff?

Is there something you wish was different or that you would like to change?

*How the Covid-19 pandemic may have affected their communication/relationship with their family members and friends:*

Can you tell us how you got information about the corona virus (Covid-19)?

Can you tell us about how the corona virus may have affected communication with the staff, relatives, and friends?

Has the pandemic/visiting restrictions affected your relationship with your relatives?

Can you tell us how you talked to your relatives during the restrictions?

*Experiences of restrictions during the Covid-19 pandemic:*

How have you been doing during the pandemic?

Do you get the care you want, or do you lack something?
